# On the Pathology of Hooping Cough

**Published:** 1856-08

**Authors:** 


*On the Pathology of Hooping Cough.*—After enumerating some of the many discrepant and imaginative theories of the nature of hooping cough that writers on the subject had indulged in, the author stated, in answer to the question. To what category of derangements do the most constant and characteristic features of the disease the most intimately unite it?—that, in his opinion, it was to the contagious fevers —to those diseases which consist of the assumption into the body of some specific *materies morbi* introduced from without, and undergoing a certain process of self-multiplication within the system—to the zymotic diseases; in fact, in favor of this view, he said, there was this threefold evidence :—

1st.—That hooping cough was contagious.

2nd.—That it runs a definite course, having certain premonitory signs: certain phenomena when the disease has attained its height, and certain sequelæ.

3rd. That it is self-prophylatic; a person having had it once, does not have it again.

Now the three circumstances—contagion, definite course, and self-prophylaxis, are, he maintained, *par excellence*, the three characteristic circumstances of the contagious fevers, and the possession by any disease of these three features would always be, to him, a sufficient warrant for its admission into that family of disorders. The author then thus stated, in more exact terms, his views: That the catching of hooping-cough depends upon the inoculation of the system with a specific poison; that this poison chooses for itself a certain eliminatory surface as its emunctory; that the surface that it so chooses is the respiratory tract of the mucous membrane, from the conjunctiva to the ultimate bronchial tubes, although the whole of the tract need not be involved in every case; that its material presence gives rise to an exalted sensibility and inflammation of the part; and that the exalted sensibility and inflammation constitute the proximate cause of the specific symptoms. The author's conviction of the correctness of the above theory was based on the following considerations:—

*a.* The premonitory symptoms of catarrh, injection of the eyes, coryza, &c.

*b.* The symptoms of vascular disturbance of the trachea, bronchial tubes, large and small, down even, in many cases, to the ultimate lung structure, that generally accompany or follow the cough.

*c.* The intermediate position in regard to time, of the laryngeal, between the nasal and the bronchial symptoms, implying a creeping down of the condition of the mucous membrane in a regular course.

*d.* The power which one child will have, who does not hoop, of communicating the disease to another who will; showing that the spasmodic part of the affection is non-essential.

*e.* The eliminatory power of the surface, which is consistent with the supposed final cause of its being affected.

*f.* The support derived from the whole weight of the argument of analogy.

Dr. Salter finished his paper by refuting, in succession, certain objections to his theory, which he could conceive others to make, but which, from our limited space, we are unable to enumerate.

Dr. Richardson believed that hooping-cough belonged to the zymotic class of diseases. He advised, that Dr. Salter should ascertain if the mucus is inoculable, and suggested the pig as a fit animal. He had seen pigs with croup, small-pox, measles, and plague. Inoculation acts well in modifying disease, by introducing but a very small dose at once, and for the same purpose, it is advantageous to inoculate from matter obtained from animals which had been the subjects of the disease.

Dr. Edward Smith had proved, in a paper published in the "Transactions" of the Royal Medical and Chirurgical Society, that the deaths from hooping-cough were mainly due to bronchitis; but he believed that inflammation was only an accident, and not an essence of the disease. He had doubts as to its being a blood disease, in the sense of being introduced into the system in the form of an organic poison; but, at all events, he considered that the spasm is all that distinguishes it necessarily from a common cough. The secretion is in great part due to the violent spasmodic cough; and the plan of treatment, which in a large experience he had found suitable, was to arrest the spasm, and thereby both the cough and the secretion; so that, in a very short time, the attack is reduced to the condition of a common cough. Since the disease may thus be cut short in probably all uncomplicated cases, and yet not be more liable to return than when allowed to run its course, he could not support the author's theory of elimination of the poison in the secretion of the mucus membrane of the larynx and bronchi. It is, however, just possible that the supposition of the gradual destruction in the system of the poison might account for the non-recurrence of the disease when thus cut short; but that would be an assumption, and, if true, would render the theory of elimination of no value in practice. He strongly commended the employment of small and increasing doses of morphia, on the plan laid down by him in a paper published in the *Edinburgh Medical Journal* for May.

Dr. Wynn believed that the disease does not run through a regular course. He admitted that it is a contagious disease, but its evidences are mainly nervons.

Dr. Webster remarked upon the difference of opinion existing as to the pathology of the disease. He did not consider it a contagious disease in the sense that measles is contagious, and he did not think it ran through a definite course. It may also recur. It is more common in the winter, and with northerly winds and frosty weather. Change of wind and air are often beneficial. Treatment will often cut short the attack. He believed the disease chiefly affected the head. It is more fatal amongst female young children.

Dr. Camps thought that the author's cases must have been complicated with some inflammatory condition. Mild temperature is beneficial. It does not run a definite course, and treatment may cut it short.

Dr. Radcliffe did not believe in the necessary connection between hooping-cough and true inflammation, and when that complication exists, the hoop is suspended. The disease is capable of being arrested, and hence does not run through a definite course.

Dr. Headland did not agree with the peripheral theory, and thought that the centric theory accounted for the production of the paroxysm. Many poisons do not act upon eliminating parts of the system. He did not approve the experimentum crucis.

The author replied.—*Proceedings of the Medical Society of London, in London Lancet.*

